# Immunoglobulin profiling with large high-density peptide microarrays as screening method to detect candidate proteins for future biomarker detection in dogs with steroid-responsive meningitis-arteritis

**DOI:** 10.1371/journal.pone.0284010

**Published:** 2023-04-10

**Authors:** Jasmin Nicole Nessler, Andrea Tipold

**Affiliations:** Department of Small Animal Medicine and Surgery, University of Veterinary Medicine Foundation, Hannover, Germany; National Research Center (NRC), EGYPT

## Abstract

Steroid responsive meningitis arteritis (SRMA) is an aberrant Th2-mediated systemic inflammatory disease in dogs. The etiopathogenesis still remains unclear as no triggering pathogen or autoantigen could be found so far. **Hypothesis**. Large high-density peptide microarrays are a suitable screening method to detect possible autoantigens which might be involved in the pathogenesis of SRMA. **Methods**. The IgA and IgG profile of pooled serum samples of 5 dogs with SRMA and 5 dogs with neck pain due to intervertebral disc herniation (IVDH) without ataxia or paresis were compared via commercially available high-density peptide microarrays (Discovery Microarray) containing 29,240 random linear peptides. Canine distemper virus nucleoprotein (CDVN) served as positive control as all dogs were vaccinated. Common motifs were compared to amino acid sequences of known proteins via databank search. One suitable protein was manually selected for further analysis with a smaller customized high-density peptide microarray. **Results**. Pooled serum of dogs with SRMA and IVDH showed different IgA and IgG responses on Discovery Microarray. Only top IgG responses of dogs with SRMA showed a common motif not related to the control protein CDVN. This common motif is part of the interleukin 1 receptor antagonist protein (IL1Ra). On IL1Ra, dogs with SRMA displayed IgA binding to an additional epitope, which dogs with IVDH did not show. **Discussion**. IL1Ra is an anti-inflammatory acute phase protein. Different immunoglobulin binding patterns on IL1Ra could be involved in the pathogenesis of SRMA and IL1Ra might be developed as future biomarker for SRMA.

## Introduction

Steroid-responsive meningitis-arteritis (SRMA) is an inflammatory disease in dogs mostly affecting the meninges and meningeal arteries of the neck [1]. Affected dogs are mostly young-adult and show signs of neck pain, fever, blood leukocytosis, and increased protein and neutrophilic pleocytosis in the cerebrospinal fluid (CSF) as well as a systemic acute phase response with increased C-reactive protein and serum amyloid-A in the serum [2, 3]. Increased levels of immunoglobulin (Ig)A can be found in serum and CSF [4]. The clinical diagnosis is achieved by combining typical clinical signs and laboratory findings after exclusion of an infectious agent [5]. The etiopathogenesis is not fully understood, yet. It is suspected that an unknown trigger causes an aberrant Th2-mediated inflammation in susceptible individuals [5]. Several studies have failed to reveal the initial trigger so far [6–9]. Therefore, the assumption prevails that after fast elimination of the presumed triggering antigen the altered immune system remains active to keep the harmful inflammation [5, 10, 11]. In human medicine, molecular mimicry is one known pathomechanism for inducing autoimmune diseases [12, 13]. Knowing the responsible triggering antigen or the equivalent cross-reacting host protein might be fundamental to completely understand the pathomechanism of SRMA and develop adequate therapy or even prevent the disease.

As a direct search for triggering antigens using various methods has so far been unsuccessful, search for immunoglobulins indicating a former host-antigen contact might be useful: Immunoglobulins formed against pathogens in the course of an infection can still be detected in the blood longtime after the actual infection and are very specific in their binding capacity [14]. Searching for immunoglobulins required knowing which triggering antigen is responsible for the disease [14]. However, using the novel technology of high-density microarrays, such knowledge is no longer necessary to find the antibodies involved. High-density microarrays allow for untargeted screening for immunoglobulin patterns [15]. Each mammal develops its own highly individualized immunoglobulin profile due to individual antigen exposure during its lifetime. These profiles overlap in patients with the same disease [16]. Overlaps could be visualized in studies using microarrays and can be used to either diagnose the disease or to help track down the presumed triggering antigen [17].

High-density microarrays are glass plates very tightly printed with different peptides [15, 18]. Approximately 35,000 different peptides fit on one glass plate. Binding of immunoglobulins to the respective peptides can be made quantifiable by fluorescence staining techniques. In this way, an exceptionally large number of possible binding possibilities can be investigated within a short time using a small sample quantity, without having to know the antibody binding epitope of the unknown antigen in advance [15]. In a second step, it is possible to further classify the antigen structure via more precise epitope searches and with the comparison of known proteins of antigens in existing databases, and thus to track down the antigen or the pathogen [19].

This technology just starts to receive attention in veterinary medicine. Initial steps included for example characterizing the immunoglobulin profile in canine lymphoma [20]. Lake et al. [21] proved that the immunoglobulin profile of dogs with meningoencephalitis of unknown origin differ from that of healthy dogs and dogs with brain tumor, each with a sensitivity and specificity of 100%, but a more detailed classification was not performed.

The following study is a pilot study to screen for possible immunoglobulin profiles in dogs with SRMA which could be used to further explore a potential candidate protein acting as autoantigen in dogs with SRMA. The detection of such a profile could help to further shed light on the etiopathogenesis of the disease and could be used as diagnostic or prognostic biomarker in SRMA.

## Overview study design

IgA and IgG immunoglobulin profiles were compared between dogs with SRMA and dogs with signs of neck pain without paresis or ataxia due to intervertebral disc herniation (IVDH).

**Fig 1:**
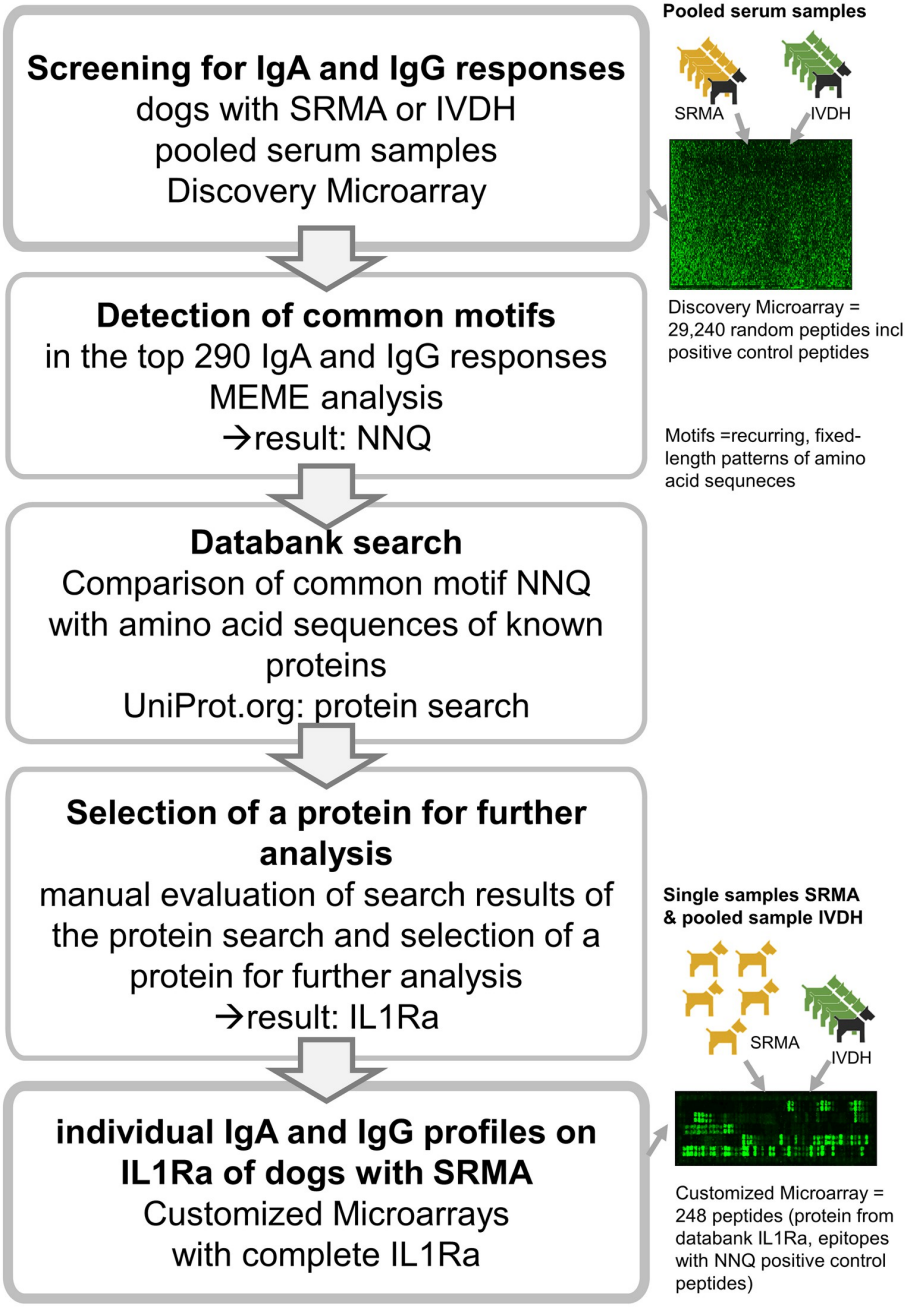
Workflow. Immunoglobulin profiles from pooled serum samples of dogs with steroid-responsive meningitis-arteritis (SRMA) or intervertebral disc herniation (IVDH) were compared via Discovery Microarray and results were analyzed for common amino acid motifs. Common motifs were compared to amino acid sequences of known proteins and printed on a second customized microarray for further analysis. Abbreviations: SRMA-steroid-responsive meningitis-arteritis; IVDH-intervertebral disc herniation; IL1Ra-interleukin 1 receptor antagonist protein; NNQ-amino acid sequence asparagine-asparagine-glutamine; Ig-immunoglobulin.

Serum samples from five dogs with SRMA and from five dogs with IVDH were pooled and the IgA and IgG immunoglobulin profile of each group was examined via large Discovery Microarray (PEPperPRINT GmbH, Heidelberg, Germany). Subsequently, epitopes with the top IgG and IgA responses of both groups were analyzed for common amino acid motifs with Multiple Expectation maximizations for Motif Elicitation (MEME) analysis (meme-suite.org [22–24]). Common amino acid motifs were compared to amino acid sequences of known proteins via databank search. After filtering and manual evaluation of the found proteins, one protein was manually chosen for further examination. This protein and all epitopes from the Discovery Microarray which contained the common amino acid motifs were used to create a second customized microarray which was used to further narrow down the IgA and IgG immunoglobulin profile of single serum samples of the dogs with SRMA. Pooled serum sample of the dogs with IVDH were used as control (Fig 1). For detailed information see the corresponding paragraph in the methods and results section.

In the following text the one-letter-abbreviations for amino acids are used according to the International Union of Pure and Applied Chemistry (IUPAC) and the International Union of Biochemistry and Molecular Biology (IUBMB) Joint Commission on Biochemical Nomenclature (JCBN) [25].

## Methods and results

### Serum samples

Archived serum samples from dogs presented to the Department for Small Animal Internal Medicine and Surgery between 2019 and 2021 with signs of neck pain caused by SRMA or IVDH were used. Dogs with IVDH were included, if they showed signs of neck pain like a stiff neck, low head carriage, spontaneous vocalization, or signs of pain on cervical palpation, no signs of paresis or ataxia, and MRI revealed a compression of the spinal cord or spinal nerves due to extradural, well demarcated T2weighted hypointense material at the level of the intervertebral disc space. SRMA was diagnosed, if the dogs were between 6 months and 8 years old, showed no signs of paresis or ataxia, but signs of neck pain like a stiff neck, low head carriage, spontaneous vocalization, or signs of pain on cervical palpation, fever (>39.5°C), blood leukocytosis (>12,000cells/μl), moderate to marked predominantly neutrophilic CSF pleocytosis (> 50cells/μl, >50% neutrophils), increased IgA in serum (>100μg/ml) and CSF (>0.2μg/ml), and no identifiable infectious agents on diagnostic work up, and responding to prednisolone treatment (1-2mg/kg once daily for 6–8 weeks followed by a tapering period). All dogs were vaccinated against canine distemper virus according to information of their owners. Blood samples were taken during routine clinical work up and used with informed owner´s consent according to university´s ethical guidelines.

Serum samples of five dog with SRMA and five dogs with IVDH were used. Mean age of dogs with SRMA was 2.1 years (range 0.5–7 years) and 9 years (range 6–12 years) of dogs with IVDH. Mean blood leukocyte count was 22.37x10³ cells/μl (range 13.8–30.6 x10³ cells/μl) in dogs with SRMA and 7.2 x10³ cells/μl (range 6.2–9.3x10³ cells/μl) in dogs with IVDH. Mean IgA content was 394.2 mg/dl (range 287.5–523.7 mg/dl) in serum of dogs with SRMA and 251.08 mg/dl (range 107.58–345.37 mg/dl) in serum of dogs with IVDH. Mean IgA content was 1.37 mg/dl (range 0.23–3.78 mg/dl) in CSF of dogs with SRMA and 0.23 mg/dl (range 0.1–0.4 mg/dl) in CSF of dogs with IVDH. Mean leukocyte count in CSF was 616.53 cells/μl (range 51–1237 cells/μl) in dogs with SRMA and 1.83 cells/μl (range 0–5 cells/μl) in dogs with IVDH. Mean percentage of neutrophilic granulocytes was 80.4% (range 72–89%) in dogs with SRMA. Cell differentiation was not performed in CSF of dogs with IVDH. Mean protein content in CSF was 180.00 mg/dl (range 31–499 mg/dl) in dogs with SRMA and 15.35 mg/dl (range 8.7–20 mg/dl) in dogs with IVDH. In all dogs with SRMA radiographs (n = 4/5) or computed tomography (n = 1/5) of the neck was performed to exclude discospondylitis. In all dogs with IVDH magnetic resonance imaging (MRI) was performed. IVDH was found three times at the intervertebral disc space of C4-5, twice at C5-6 and once T9-10. One dog had IVDH at two sides.

### Immunoglobulin profiling with discovery microarray

Immunoglobulin profiling for IgA and IgG of both groups was performed using 3D printed high-density peptide microarrays on glass slides [15, 18, 26], which were commercially available PEPperCHIP^®^ Signature Discovery Microarrays (PEPperPRINT GmbH, Heidelberg, Germany) with mild modifications. Discovery Microarrays contain 29,240 random linear 15 aa peptides with an amino acid frequency of 5% for A, D, E, F, G, H, I, K, L, M, N, P, Q and V and 6% for R, S, T, W and Y printed in duplicate (58,480 peptide spots). The arrays were customized by addition of canine distemper virus nucleoprotein (CDVN, UniProt.org ID: P04865 [27]) converted into 262 different 15 amino acid peptides with a peptide-peptide overlap of 13 amino acids printed in duplicate (524 peptide spots) as positive control. The Discovery Microarrays were further framed by additional control peptides: influenza virus antigen hemagglutinin (HA) epitopes (YPYDVPDYAG, 264 spots), poliomyelitis virus (polio, KEVPALTAVETGAT, 257 spots,) and c-Myc (EQKLISEEDL, 260 spots) peptides (S1 Table) as internal quality control to confirm the assay quality and the peptide microarray integrity [28].

For the first analysis 50 μl of each serum of all five dogs in each group were pooled and for each group one discovery microarray was used. Processing was performed as described before [28–30] with the following secondary antibodies: Goat anti-dog IgG (Fc) DyLight680 (0.4 μg/ml), goat anti-dog IgA DyLight800 (1.0 μg/ml), and monoclonal mouse anti-HA (12CA5) DyLight680 (all Rockland Immunochemicals Inc., Pottstown, PA, USA).

A peptide microarray was pre-stained with the secondary antibodies in incubation buffer to investigate background interactions with the peptides of the microarray that could interfere with the main assays. No background interaction of the secondary goat anti-dog IgG (Fc) DyLight680 immunoglobulin in the red channel was observed that could interfere with the main assays. Multiple background interactions of the secondary goat anti-dog IgA DyLight800 immunoglobulin was observed in the green channel; these background interactions were quantified and are given separately in S1 Table. Due to mostly low signal secondary antibodies background interactions were not further considered for the rest of the experiment.

Quantification of spot intensities was based on the 16-bit gray scale tiff. Microarray image analysis was done with PepSlide^®^ Analyzer (LI-COR Odyssey Imaging System; scanning offset 0.65 mm, resolution 21 μm, scanning intensities of 8/8 (red = 700 nm/green = 800 nm; PEPperPRINT GmbH, Heidelberg, Germany)) and summarized in Excel files (Microsoft Corporation, Redmond, Washington, USA). A software algorithm breaks down fluorescence intensities of each spot into a raw signal of which the global background signal (= median of all the intermediate spot areas) was subtracted from the on-spot values to calculate the foreground signal, averaged median foreground intensities and spot-to-spot deviations of spot. Based on averaged median foreground intensities, intensity maps were generated and interactions in the peptide maps highlighted by an intensity color code with red (IgG) or green (IgA) for high and white for low spot intensities [31]. Maximum spot-to-spot deviation of 40% was tolerated, otherwise the corresponding intensity value was set to zero as recommended by the manufacturer [29, 32].

To identify the top IgG and IgA responses of the canine serum pools, the averaged and corrected intensity values were sorted by decreasing spot intensities. Averaged spot intensities of the assays with the canine serum pools were further plotted against the microarray content from top left to bottom right of the microarray to visualize overall spot intensities and signal-to-noise ratios.

Clear IgG and IgA response were visible against epitope-like spot patterns formed by adjacent peptides with a consensus motif among the CDVN peptides, differing for both samples (S1 Table).

Following the assays at a dilution of 1:100, staining with the control anti-HA immunoglobulin gave rise to the expected well-defined HA control spot pattern framing the peptide microarrays, validating the overall peptide microarray integrity and assay performance. No response was visible against the negative control epitopes of polio and c-Myc (data not shown).

The IgG and IgA responses exhibited different clear and well-defined spot pattern to different peptides in both groups (S1 Table).

Top 1% IgA and IgG response were chosen for further motif discovery. The lowest signal intensity of the top 1% IgA response in SRMA responses presented with fluorescent intensity of 2740.5 fluorescence arbitrary units and 1612.8 fluorescence arbitrary units in IVDH, respectively. The lowest signal intensity of the top 1% IgG responses presented with a signal intensity of 3274.3 fluorescence arbitrary units in SRMA and 1346.8 fluorescence arbitrary units in IVDH, respectively (S1 Table).

### Motif discovery

To identify a common motif among the top IgG and IgA responses of the canine serum pools, MEME bioinformatic analyses was performed of the top 290 (1%) responses including the CDVN epitopes (meme-suite.org [22–24]). The top peptides of each pool were uploaded to the MEME tool for discovering motifs in a group of related protein sequences. MEME represents motifs as position-dependent letter-probability matrices which describe the probability of each possible letter at each position in the pattern [23, 24].

The MEME pre-settings were a maximum of 5 different motifs per analysis, one motif per peptide. In the MEME output, the E-value corresponds to the statistical significance of a consensus motif with the given log likelihood ratio (or higher), and with the same width and site count, that one would find in a similarly sized set of random sequences. An E-value of 1 would be expected for the identification of a certain motif in a set of random formed peptides; decreasing E-values correlate with increasing statistical significance. A significance threshold of E < 5.0e-002 was defined for this analysis.

The MEME analysis of the top 290 IgA responses of dogs with IVDH did not highlight any common motifs with a statistical significance. MEME analysis of the top 290 IgA responses of dogs with SRMA highlighted one common motif attributed to the CDVN control peptide (TAPYMVIL; E 2.9e-003).

MEME analysis of the top 290 IgG responses of dogs with IVDH highlighted four common motifs attributed to the CDVN control peptides: DIDNY (E 2.7e-015), ERLPGYTP (E 2.8e-008), WSESRYDTQII (E 2.7e-006), and DGNDDDRK (E 2.3e-004). MEME analysis of the top 290 IgG responses of dogs with SRMA highlighted three common motifs attributed to the CDVN control peptides: RYDTQIIQ (E 1.5e-012), DDGNDDDRKSM (E 2.7e-007), and QGGDKYPIHFS (E 4.0e-003). Additionally, MEME analysis highlighted the common motif NNQ (E 3.0e-002), which is not attributed to any control epitope and occurred in 26 of the 290 top IgG responses of dogs with SRMA.

### Comparison of common motif NNQ to amino acid sequences of known proteins

The common motif NNQ found in the top IgG responses of dogs with SRMA was searched in amino acid sequences of known proteins. A peptide search in uniprot.org was performed [33]. Only search results reviewed by UniProt (Swiss-Prot; UniProt.org [27]) were considered for further analysis to avoid redundancy and low level of evidence. First search was not restricted by taxonomy and included all organisms. Second search restricted results to the dog (*canis lupus familiaris*). The search results were manually evaluated, and a possible candidate protein was chosen for further evaluation.

NNQ was found as part of 16,229 proteins of several lifeforms, for example *Sus scrofa* (pig), *Xenopus laevis* (African clawed frog), *Synanceia verrucosa* (Reef stonefish), *Acinetobacter baumannii*, Aspergillus spp., *Campylobacter jejuni*, *Clostridium botulinum*, or *Drosophila melanogaster* (uniprot.org, search ID: PM2022111647fffabf2b844ea9a5f3542dd9240009 https://www.uniprot.org/peptide-search/PM2022111647fffabf2b844ea9a5f3542dd9240009/overview). In dogs (*canis lupus familiaris*), 21 proteins were found that contain NNQ (uniprot.org, search ID: PM20221116faeb6e4d2a474bc2a8a32fca666edb3e, https://www.uniprot.org/peptide-search/ PM20221116faeb6e4d2a474bc2a8a32fca666edb3e /overview; Table 1). Amongst others, Interleukin 1 receptor antagonist protein (IL1Ra) was found and manually chosen for further analysis.

**Table 1:** In dogs (*canis lupus familiaris*), 21 proteins were found that contain NNQ data bank search: https://www.uniprot.org/peptide-search/PM20221116faeb6e4d2a474bc2a8a32fca666edb3e/overview.

UniProt.org Entry No ID	Match, position within the protein	Protein Names
Q9TT90	Positions 679–681: NNQ	Androgen receptor
Q9N2J4	Positions 41–43: NNQ	Aquaporin-1
P09582	Positions 180–182: NNQ	Arginine esterase
Q5D0K2	Positions 189–191: NNQ	Cholecystokinin receptor type A
Q5XWD5	Positions 319–321: NNQ	Cobalamin binding intrinsic factor
O46392	Positions 1143–1145: NNQ	Collagen alpha-2(I) chain
Q9TU53	Positions 1640–1642: NNQ	Cubilin
Q28275	Positions 1932–1934: NNQ	Fibronectin
E2QYC9	Positions 1180–1182 & 1293–1295: NNQ	InaD-like protein
Q9BEH0	Positions 52–54: NNQ	Interleukin 1 receptor antagonist protein
Q28275-1	Positions 1927–1929: NNQ	Isoform 1 of Fibronectin
Q28275-3	Positions 1927–1929: NNQ	Isoform 2 of Fibronectin
Q6EIY9	Positions 189–191: NNQ	Keratin, type II cytoskeletal 1
Q2TLZ1	Positions 374–376: NNQ	Macoilin
O46501	Positions 173–175: NNQ	Major prion protein
F1PT61	Positions 68–70: NNQ	Myosin-16
Q28895	Positions 134–136: NNQ	NPC intracellular cholesterol transporter 2
Q8MJ04	Positions 400–402: NNQ	Oxygen-regulated protein 1
Q9GMY6	Positions 377–379: NNQ	Pepsin A
O18735	Positions 176–178 & 318–320: NNQ	Receptor tyrosine-protein kinase erbB-2
F1PRN2	Positions 437–439: NNQ	Unconventional myosin-Id

Abbreviations: No ID-identification number; NNQ-amino acid sequence asparagine-asparagine-glutamine.

### Customized microarray with IL1Ra and NNQ motifs

After choosing IL1Ra from the protein data bank search for further evaluation, it was printed on a high-density customized microarray (possible up to 500 spots): The whole sequence of canine IL1Ra (UniProt.org ID: Q9BEH0 [27]) was linked and elongated with neutral GSGSGSG linkers at the C- and N-termini to avoid truncated peptides. The elongated antigen sequence was converted into linear 15 amino acid peptides with a peptide-peptide overlap of 14 amino acids. In addition, the microarray contained all peptides of the previous Discovery Microarray which contain all 49 the NNQ motif, and 23 CDVN-related peptides, which showed positive IgA or IgG response in the previous Discovery Microarray. The resulting linear peptide microarrays contained 248 different peptides printed in duplicate (498 spots) and were framed by additional HA (YPYDVPDYAG, 62 spots) control peptides. Processing was the same as for the Discovery Arrays.

CDVN epitopes exhibited up to moderate (3001–12,000 fluorescence arbitrary units) IgA and high IgG spot intensities in all dogs with SRMA and in pooled serum of dogs with IVDH. NNQ peptides exhibited up to strong (>12,000 fluorescence arbitrary units) IgG and IgA spot intensities in all samples, especially in dog 2 with SRMA, while in the other dogs with SRMA and the pooled sample of dogs with IVDH, the NNQ motifs showed mostly mild (500–3000 fluorescence arbitrary units) to moderate intensity levels (S2 Table).

IgG responses in all dogs with SRMA and pooled control serum of dogs with IVDH were directed against epitope-like spot patterns formed by adjacent peptides with the consensus motifs RPCRMQAFRIWDVN and DVNQKTFYLRNNQ of IL1Ra. Especially dog 2 with SRMA showed high IgG response against DVNQKTFYLRNNQ, which contains the common motif NNQ (S2 Table).

**Fig 2:**
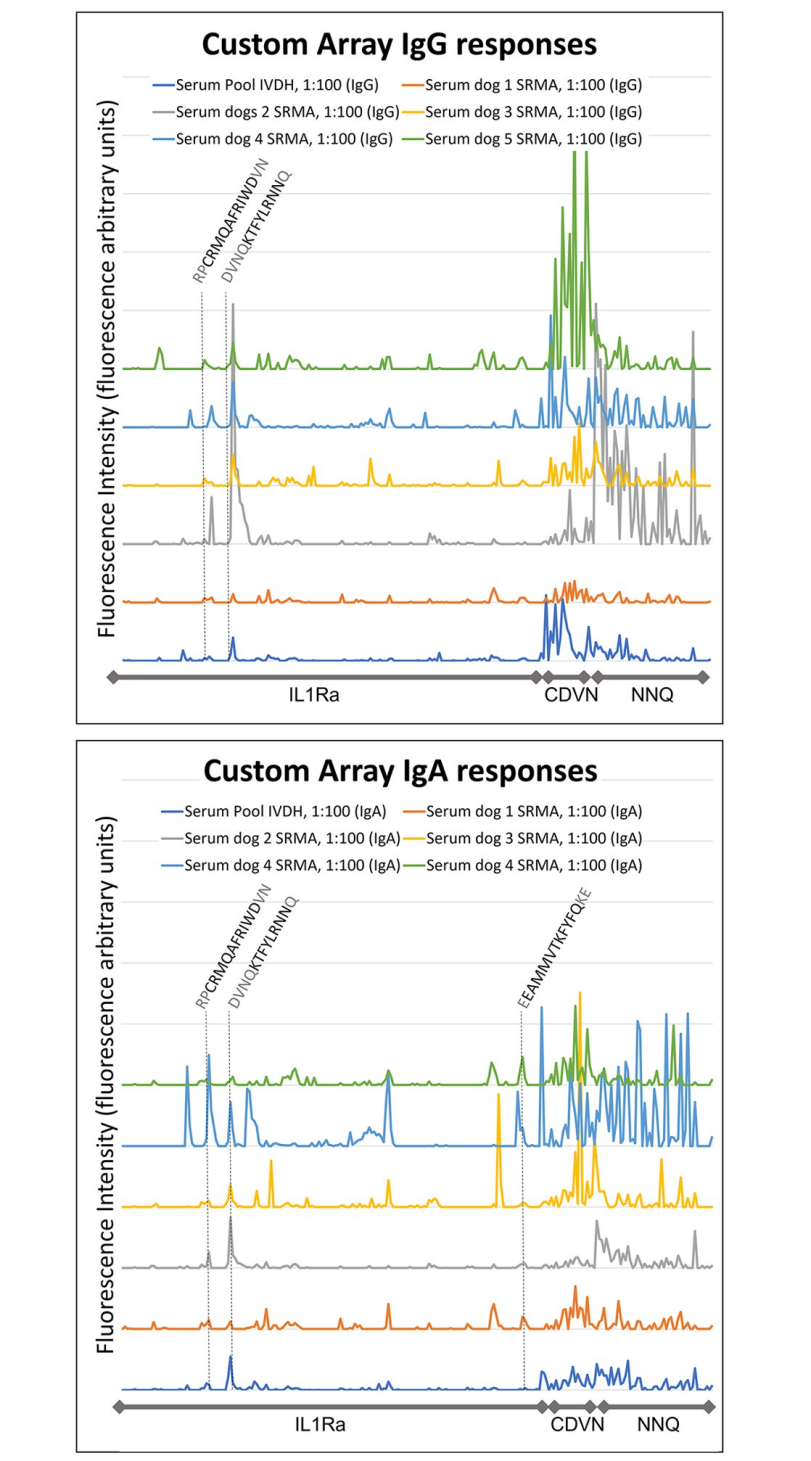
Immunoglobulin binding response of dogs with steroid responsive meningitis-arteritis (SRMA) and intervertebral disc herniation (IVDH) in the customized microarray. Each horizontal line represents 10,000 fluorescence arbitrary units. For a better data overview, the baseline of each sample were shifted up. Abbreviations: Ig-immunoglobulin; IL1Ra-interleukin 1 receptor antagonist protein, CDVN-epitopes from nucleoprotein of canine distemper virus, NNQ-motifs containing the amino acid sequence asparagine-asparagine-glutamine.

IgA responses in all dogs with SRMA and pooled control serum of dogs with IVDH were directed against epitope-like spot patterns formed by adjacent peptides with the consensus motifs RPCRMQAFRIWDVNQ and DVNQKTFYLRNNQ. In addition, all dogs with SRMA exhibited shared IgA immunoglobulin responses against epitope-like spot patterns with the consensus motif EEAMMVTKFYFQKE (Fig 2).

## Conclusion

**Fig 3:**
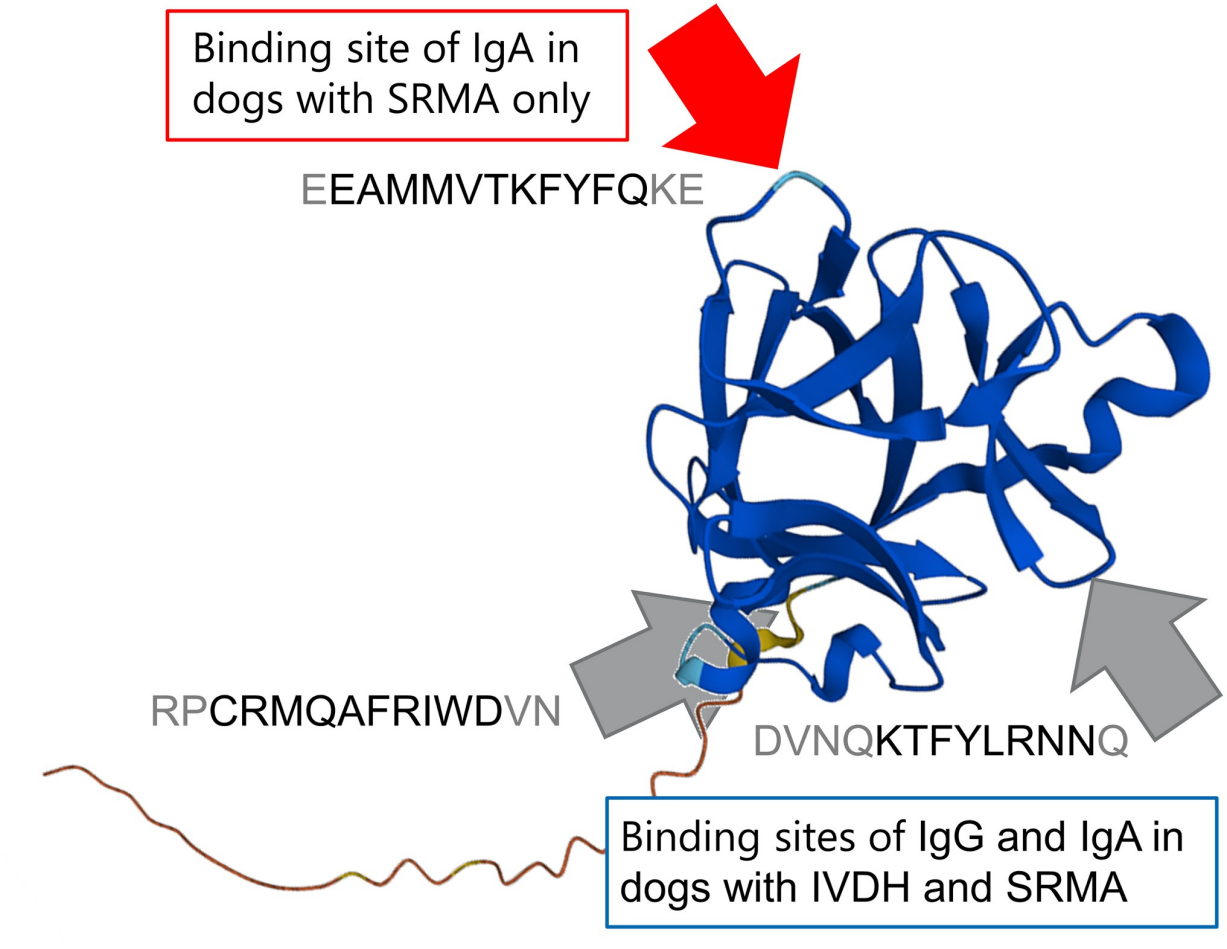
Protein structure of canine Interleukin 1 receptor antagonist protein (IL1Ra) (modified after https://alphafold.ebi.ac.uk/entry/Q9BEH0 [34]) with IgG and IgA binding epitopes. Abbreviations: SRMA-steroid-responsive meningitis-arteritis; IVDH-intervertebral disc herniation; Ig-immunoglobulin; EEAMMVTKFYFQKE, RPCRMQAFRIWDVN, DVNQKTFYLRNNQ—amino acid sequences [28].

Summarizing, we found similar binding pattern on IL1Ra of IgA and IgG of dogs with SRMA and pooled serum samples of dogs with IVDH with varying intensity levels, but all five dogs with SRMA showed additional IgA binding against EEAMMVTKFYFQKE of IL1Ra, which dogs with IVDH did not exhibit (Fig 3).

## Discussion

In the current manuscript the usage of high-density peptide microarrays as screening method for immunoglobulin profiling in dogs with SRMA is presented, which could help to detect diagnostic or prognostic biomarkers as well as help to uncover the etiopathogenesis of SRMA. We identified IL1Ra as a candidate protein which could potentially be involved in the pathogenesis of SRMA, might act as autoantigen and showed that IgA binding is different on IL1Ra in dogs with SRMA compared to the control dogs.

Pooled serum samples of dogs with SRMA and dogs with IVDH showed different common IgA and IgG responses on the Discovery Microarray. Both groups displayed IgG responses against epitopes which are part of the CDVN protein. CDVN was chosen as positive control protein as all dogs were vaccinated against canine distemper virus. Such a vaccination induces immunoglobulin responses against the nucleoprotein of the virus [35]. In contrast, only in dogs with SRMA a common IgA motif attributed to CDVN was detected. This could be due to the fact that IgG persists longer in the circulation, while IgA has a shorter half-lifetime [36]. Therefore, a significant amount IgA related to CDV vaccination might be eliminated at the time of our examination in the older dogs with IVDH. In contrast to dogs with IVDH, dogs with acute SRMA are experiencing an unspecific increase of IgA [4], which also includes CDV vaccination related IgA. Therefore, IgA response to CDVN might be detectable despite absence of acute CDV infection or vaccination, while it is not detectable in dogs with IVDH.

This manuscript describes a first screening attempt with large high-density peptide to detect possible autoantigens which might be involved in the pathogenesis of SRMA. In the Discovery Microarray, only in the pooled sample of dogs with SRMA an additional IgG response against a common motif unrelated to CDVN was found: NNQ. Several technical details might be taken into account when interpreting these data. The peptide microarrays contained only linear epitopes, where a sequence of continuous amino acids are sufficient for antibody binding. On the contrary, conformational epitopes are born out of the tertiary protein structure and antibody binding sides are formed by connecting distant amino acids by protein folding (reviewed by Forsström [37]). Antibodies binding on conformational epitopes only might be missed with the linear peptides.

Using pooled samples is cost and time efficient and might therefore considered to be suitable for a first screening attempt for biomarkers [38, 39]. Follow up investigations might be needed as pooled samples could lead to divergent results in one of the following scenarios: One single patient with high amount of a specific antibody might mimic a common motif although the serum of other patients do not contain this antibody. On the other hand, serum of patients who do not display this antibody might dilute the antibody concentration in the pooled sample to a point where the antibody cannot be detected anymore or it might not fall within the top 1% of antibody binding responses and the epitope might not be considered significant and therefor be missed.

Choice of control group might also have an impact on results [40]. Dogs with IVDH were preferred as control group over healthy dogs in the present study to minimize false positive findings because clinical signs and basic immunological activation due to pain related stress might be similar. In contrast to SRMA, the pathogenesis of IVDH is not primary inflammatory (reviewed by Olby et al. [41]). Additionally, private client owned dogs were preferred over clinic owned healthy dogs as control. Those dogs are disposed to a more complex antigenetic environment and therefore comparable to client owned dogs with SRMA. In contrast, clinic owned animals live in a more sanitary environment with less and different antigen contact [42, 43] leading to an immunoglobulin profile diverging per se from “free-roaming” client owned dogs.

Mean age between groups differ because SRMA is known to occur mostly in young adult dogs, while dogs with IVDH are mostly older [2, 3, 41]. Although in human medicine, matching control samples in age and gender seems to be advised [44] for biomarker detection, it seems this is less crucial in canine immunosignature [21] and in several studies, no difference in in vivo antibody responses was seen between older and young dogs [45, 46].

NNQ is a very short motif and can be found in more than 16,000 different proteins from a variety of organisms, for example mammals, fishes, frogs, worms or bacteria (https://www.uniprot.org/peptide-search/PM2022111647fffabf2b844ea9a5f3542dd9240009/overview). The high number of candidates for possible triggers makes the NNQ motif as part of a common infectious trigger unlikely. Therefore, the present study focused on candidates for potential canine autoantigens in proteins with NNQ motif being part of the patient’s organism. Of 21 possible host related proteins, one seemed most likely be involved in the pathogenesis of SRMA: Interleukin 1 receptor protein (IL1Ra). IL1Ra is an acute phase protein and is upregulated together with IL-1, with IL1Ra being an anti-inflammatory antagonist of IL1 receptors [3, 47, 48]. SRMA is known to be a Th2-mediated systemic inflammatory disease with marked increase of acute phase proteins as CRP [2, 3] and with upregulation of proinflammatory cytokines [4]. Therefore, it is reasonable to suggest that IL-1 and IL1Ra might be involved in its pathogenesis.

On the customized microarray the dogs with SRMA showed almost the same immunoglobulin profile against IL1Ra as the pooled serum sample from dogs with IVDH. It seems that in both groups IgG and IgA against IL1Ra were present and it could be concluded, that this phenomenon is attributed to diseases affecting the meninges or might be normal in all dogs. However, all dogs with SRMA displayed an additional IgA response against IL1Ra on another epitope of the protein, which was not observed in the pooled serum of dogs with IVDH. IL1Ra is a protein of the IL-1 family with a length of 176 amino acids (UniProt.org ID: Q9BEH0 [30]). It is produced by many different cells, for example immune cells, epithelial cells, or stromal cells (reviewed by Perrier et al. [49]). It is an antagonist of the proinflammatory cytokines IL-1α and IL-1β by competitive antagonization of the IL-1 receptor [49, 50]. IL1Ra is therefore an anti-inflammatory acute-phase protein [49]. An IL-1-IL1Ra-dysbalance in human medicine is attributed to several diseases like sepsis, neoplasia, or several autoimmune diseases [49, 51–53]. Additionally, a more severe clinical course in several inflammatory diseases like osteoarthritis, gingivitis, or COVID-19 is correlated to one of four different genetic polymorphisms of IL1Ra [54–56]. Inborn deficiency of IL1Ra results in sever autoimmune disease involving bone and skin in children [57].

A different immunoglobulin binding profile against IL1Ra in dogs with SRMA compared to dogs with IVDH might be caused by an unspecific increase of IgA production or might just be an expression of increased IL1Ra production in the course of acute SRMA. On the other hand, in the pathogenesis of SRMA, IL1Ra could either develop a different conformation exposing an additional binding site for immunoglobulins or dogs with SRMA do have an inherited divergent IL1Ra confirmation and subsequent tertiary structure, which might induce an immunoglobulin response on the additional binding side in dogs with SRMA. Another possibility might be, that the initial triggering antigen also expresses this epitope leading to cross-reaction of IgA with this additional binding epitope on IL1Ra. A different conformation of IL1Ra could make the protein less effective reducing the proinflammatory reaction, or immunoglobulins against IL1Ra could lower the efficacy of the protein and therefore cause or aggravate the course of the inflammation in SRMA.

Summarizing, high-density discovery arrays are a suitable and fast screening method for potential candidates for biomarker or possible research on the etiopathogenesis of SRMA. Additionally, high-density microarrays can detect different immunoglobulin binding profiles against different epitopes within a possible candidate protein which might suggest the assumption that different conformation stages of IL1Ra could be involved in the course of SRMA, which needs further examination of the tertiary protein structure. Different IgA binding profiles of dogs with SRMA compared to pooled serum sample of dogs with IVDH could be the corner stone for further research and might help discover the etiopathogenesis of SRMA and might even be a groundbreaker to develop new therapeutic strategies in the future.

## Supporting information

S1 Table Amino acid sequences of epitopes on the Discovery Microarrays and fluorescent intensity (fluorescence arbitrary units) of the IgA and IgG response of pooled samples from dogs with steroid-responsive meningitis-arteritis (SRMA) or intervertebral disc herniation (IVDH) as well as of the secondary immunoglobulin goat anti dog IgA, which gave mild to moderate signal. The table shows averaged spot intensities of the assays with the canine serum pools against the microarray content and the calculation is based on the corrected intensity values. The position on the array is given in “row” and “column”. The epitope is given as one-letter abbreviation of amino acids [28]. Fluorescence intensity is given in fluorescence arbitrary units for the IgA and IgG responses of the pooled serum samples of dogs with SRMA or IVDH and for the corresponding secondary antibodies. Abbreviations: Ig-immunoglobulin; CDV- canine distemper virus.
(XLSX)

S2 Table Amino acid sequences of epitopes on the Customized Microarray and the IgA and IgG response fluorescent intensity (fluorescence arbitrary units) of individual samples from dogs with steroid-responsive meningitis-arteritis (SRMA) or pooled samples from dogs with intervertebral disc herniation (IVDH). The table shows averaged spot intensities of the assays with the single canine serum samples and the pooled serum against the microarray content and the calculation is based on the corrected intensity values. The position on the array is given in “row” and “column”. The epitope is given as a one-letter abbreviation of amino acids [28]. Fluorescence intensity is given in fluorescence arbitrary units for the IgA and IgG responses of the serum samples. Abbreviations: Ig-immunoglobulin; IL1Ra-interleukin 1 receptor antagonist protein, CDVN-nucleoprotein of canine distemper virus.
(XLSX)
